# Community attitudes on genetic research of gender identity, sexual orientation, and mental health

**DOI:** 10.1371/journal.pone.0235608

**Published:** 2020-07-08

**Authors:** Taylor R. Thomas, Dabney Hofammann, Brooke G. McKenna, Anna I. R. van der Miesen, Mark A. Stokes, Peter Daniolos, Jacob J. Michaelson

**Affiliations:** 1 Department of Psychiatry, University of Iowa, Iowa City, Iowa, United States of America; 2 Department of Psychology, Emory University, Atlanta, Georgia, United States of America; 3 Department of Child and Adolescent Psychiatry, Amsterdam UMC, Amsterdam, North Holland, Netherlands; 4 Department of Psychology, Deakin University, Melbourne, Victoria, Australia; Hunter College, UNITED STATES

## Abstract

Sex is an important factor in mental health, and a non-binary view of how variation in sex and gender influence mental health represents a new research frontier that may yield new insights. The recent acceleration of research into sexual orientation, gender identity, and mental health has generally been conducted without sufficient understanding of the opinions of sexual and gender minorities (SGM) toward this research. We surveyed 768 individuals, with an enrichment of LGBTQ+ stakeholders, for their opinions regarding genetic research of SGM and mental health. We found that the key predictors of attitudes toward genetic research specifically on SGM are 1) general attitudes toward genetic and mental health research 2) tolerance of SGM and associated behaviors and 3) age of the participant. Non-heterosexual stakeholder status was significantly associated with increased willingness to participate in genetic research if a biological basis for gender identity were discovered. We also found that heterosexual, cisgender participants with a low tolerance for SGM indicated their SGM views would be positively updated if science showed a biological basis for their behaviors and identities. These findings represent an important first step in understanding and engaging the LGBTQ+ stakeholder community in the context of genetic research.

## Introduction

Biological sex (see Table 1 for definitions) interacts with other risk factors in ways that are often strongly predictive of health outcomes. From immune response [1] to heart disease [2] and depression [3], sex is a key variable that contextualizes risk factors [4] and can yield biological insights into disease mechanisms. What is less understood is whether a continuous (rather than binary) view of sex and gender can deliver additional explanatory power in studies of human health and disease. Involving sexual and gender minorities (SGM, or more commonly referred to as the LGBTQ+ community—lesbian, gay, bisexual, transgender, and queer) can provide an important view of gender and sexuality that is not restricted to a strictly binary perspective when considering sex differences in human health. Of particular interest are neuropsychiatric conditions that have strong sex biases and have shown evidence of enrichment for non-heterosexuality and gender variance. For instance, autism is highly male-biased (4:1) [5] and is also enriched for gender dysphoria [6, 7], while anorexia nervosa is highly female-biased (8:1) [8] but is enriched for gay and bisexual men [9]. More generally, there has been extensive study into the higher prevalence of neuropsychiatric conditions in the LGBTQ+ community [10]. While it is possible that the increased prevalence of neuropsychiatric conditions in the LGBTQ+ community are due primarily to sociocultural factors like prejudice, stigma, discrimination, and rejection [11], few studies have investigated potential biological factors underlying these connections, especially working with the understanding that sex and gender are more complex, continuous factors.

**Table 1:** Key definitions for terms used.

Definitions	
Biological sex	sex as directed by genetic factors, that then determine downstream sex-differentiating biological processes
Recorded sex	sex recorded at time of birth based on physiological and anatomical sex characteristics; has also been referred to as natal sex or assigned sex
Transgender	an umbrella term that describes an individual who does not identify or exclusively identify with their recorded sex
Cisgender	term describing an individual who identifies with the gender that is consistent with their recorded sex
Gender	the behavioral norms for each sex that emerge at the population level, driven by a combination of biological, social, and cultural influences
Gender identity	the relation of an individual to gender norms that is most consistent with that individual’s feelings, perspectives, and behaviors
Gender expression	the way in which an individual shows their gender identity through physical appearance, behavior, and interests
Sexual orientation	a component of an identity that includes a person’s sexual and emotional attraction to another person, as well as the behavior and/or social affiliation that may result from this attraction
Non-heterosexuality	an encompassing sexual identity term for those who are not strictly heterosexual
Gender variance	the discrepancy between an individual’s gender identity and the gender typical of their recorded sex
Sexual and gender minorities (SGM)	an encompassing term for people who are not heterosexual and/or not cisgender
Stakeholder	an individual who has an investment in a group and is affected by their involvement in the group

In August 2019, the first large-scale study of same-sex sexual behavior appeared in *Science* [12]. As the tools for genomic research have become more accessible, fields beyond medicine, including social science, are increasingly appealing to genetic data in the search for explanatory factors of human behavior and identities. This trend reflects an entry into the “genomics to society” phase of a tripartite goal laid out in 2003 by the National Human Genome Research Institute [13]. With this transition comes an urgent need to understand the perspectives and concerns of both the general public and the groups being studied. This is particularly true in the case of sexual orientation [14], gender identity, and their potential connections to aspects of mental health, which have received increasing attention from genetic researchers in recent years [15–17]. While genetics cannot fully explain these sensitive and often stigmatized [18] aspects of individual identity, a greater understanding of the genetic and biological contributions to these phenomena may reduce public stigma while also advancing scientific understanding of the complex relationships between sex, gender, and risk for neuropsychiatric conditions. This dual potential can only be achieved through partnership between scientists and the LGBTQ+ stakeholder community. Therefore, the objective of this exploratory study was twofold: first, to obtain a systematic, data-driven assessment of attitudes related to genetic research of sexuality and gender identities, and secondly to give a voice in the scientific literature to stakeholder groups and use their opinions to help inform the research that affects them. Our working hypothesis was LGBTQ+ stakeholders (as defined by those who reported a non-cisgender identity or non-heterosexual orientation) would be less likely to positively endorse genetic research into sexual orientation and gender identity.

## Materials and methods

This study was approved the University of Iowa’s Institutional Review Board (IRB #201611784). The survey was built on the Qualtrics platform.

### Participants

Participants were primarily recruited through mass email to the University of Iowa, as well as through social media. Participants had to be 18 years or older to be eligible for participation in the study. Participant demographics are presented in Table 2. The main terms used for the demographic descriptions for sex, sexual orientation, and gender identity are provided in Table 1.

**Table 2:** Counts and percentage of participants who endorsed a demographic characteristic. For gender identity, sexual orientation, race, and religious affiliation, individuals were able to endorse multiple identities. For example, the participant could have selected bisexual, pansexual, and queer, which contributes a count to each of those three categories.

Characteristics of Participants					
**Gender identity**	**Counts**	**Percent**	**Recorded sex**	**Counts**	**Percent**
Cisgender	683	89%	Female	592	77%
Transgender	37	5%	Male	171	22%
Non-binary	33	4%	Intersex	2	0%
Gender neutral	26	3%	Other	3	1%
Genderqueer	25	3%	**Race**	**Counts**	**Percent**
Demigender	24	3%	White or Caucasian	717	93%
Gender fluid	18	2%	Asian	31	4%
Agender	14	2%	Hispanic or Latino	29	4%
Pangender	6	1%	Black or African American	15	2%
Third gender	5	1%	Native American or Alaskan Native	8	1%
Bigender	2	0%	Native Hawaiian or other Pacific Islander	1	0%
Other	19	2%	Other	6	1%
Not sure	5	1%	Prefer not to say	4	1%
**Sexual orientation**	**Counts**	**Percent**	NA	2	0%
Heterosexual	498	65%	**Religious affiliation**	**Counts**	**Percent**
Bisexual	145	19%	Christian	342	45%
Queer	84	11%	Non-religious	153	20%
Gay	71	9%	Atheist	113	15%
Pansexual	65	8%	Agnostic	107	14%
Lesbian	50	7%	Spiritual	103	13%
Gray asexual	50	7%	Jewish	19	2%
Monosexual	40	5%	Hindu	8	1%
Homosexual	38	5%	Buddhist	5	1%
Asexual	34	4%	Muslim	4	1%
Polysexual	23	3%	Other	26	3%
Other	20	3%	Prefer not to say	9	1%
Not sure	12	2%	NA	1	0%
Prefer not to say	1	0%	**Annual income**	**Counts**	**Percent**
**Age in years**	**Counts**	**Percent**	Less than $14,999	254	33%
18-29	409	53%	$15,000—$34,999	123	16%
30-39	99	13%	$35,000—$49,999	95	12%
40-49	88	11%	$50,000—$74,999	124	16%
50-59	78	10%	$75,000—$99,999	63	8%
60+	50	7%	$100,000—$199,999	81	11%
**Educational attainment**	**Counts**	**Percent**	More than $200,000	20	3%
High school	45	6%	NA	8	1%
Associate’s degree	52	7%	**Developed environment**	**Counts**	**Percent**
Some college, no degree	251	33%	Urban	252	33%
Bachelor’s	186	24%	Suburban	366	48%
Master’s	121	16%	Rural	118	15%
Professional degree	27	4%	Other	16	2%
Doctorate	82	11%	Not sure	14	2%
Prefer not to say	1	0%	NA	2	0%
NA	3	0%			

### Procedures

After participants indicated interest and consented to the survey, they were able to complete the Qualtrics survey. The complete survey is available in S1 File.

### Measures

#### Online survey

The survey was designed to capture the participant’s knowledge and views on genetic research broadly, as well as genetic research into mental health, neuropsychiatric conditions, sexual orientation, and gender identity. We also asked questions regarding the participant’s opinions on non-heterosexuality and non-cisgender identities. Opinion data was collected on a 5-point Likert scale. Considering our main goal was to understand the opinions of people in specific communities, we collected in-depth data regarding their own sexual orientation, gender expression, and gender identity. In addition, we collected basic demographic information. Demographic data for sexual orientation, gender identity, religion, race, and ethnicity were collected with the participant able to select multiple values to describe themselves. A demographic summary is displayed in Table 2. Survey participants were also asked to arrange continuously-adjustable sliders (representing femininity, masculinity, and “other”, which they could name themselves in a free text box) in a way that best described their gender identity. At the end of the survey, participants were asked open-ended, free text questions in which they were able to detail their opinions and concerns, and were given the option to provide contact information to be contacted about future research opportunities.

#### Gender space and continuous-valued gender variance

In addition to providing categorical descriptors of gender identity that participants could choose to endorse, we provided three continuously-adjustable sliders (0-1 for each of femininity, masculinity, and “other”, which participants could re-name if they wished), and asked participants to arrange the sliders in a way they felt was most consistent with their gender identity. The values from these sliders comprise a three-dimensional gender space, where each participant can be described by a triplet coordinate of [femininity, masculinity, other] (Fig 2). We calculated a scalar-valued gender variance score by taking each participant’s gender coordinates compared to their recorded sex (sex recorded at time of birth), and calculating the Euclidean distance to a gender datum of [1, 0, 0] if the participant reported being a recorded female, and [0, 1, 0] if the participant reported being a recorded male. For this particular analysis, those who reported intersex as their recorded sex (*N = 2*) were excluded.

#### Tolerance indicator

In order to evaluate how overall tolerance of non-heterosexuality and gender variance influenced opinions towards genetic research of sexual orientation and gender identity, we developed a tolerance indicator. This was built using Likert scale responses to the statements presented in Table 3. To facilitate a grouped analysis of tolerance as it relates to other measures, we binned participants into three groups, which we labeled as “intolerant” (bottom quartile), “moderately tolerant” (interquartile range) and “tolerant” (top quartile).

**Table 3:** Participants answered on a 5-point Likert scale to the following statements which were used to build a non-heterosexuality and gender variance tolerance indicator by multiplying by the indicated score (+1 for higher tolerance and -1 for lower tolerance).

Statements used to build tolerance indicator	Score
Women should only be attracted to men.	-1
It is okay that people dress in ways that don’t conform with their sex assigned at birth.	+1
People are supposed to be male or female.	-1
Men should only be attracted to women.	-1
Some people do not experience sexual attraction at all.	+1

### Statistical analyses

#### Imputation of missing data

Tabular survey data was extracted from Qualtrics and analyzed in R. Surveys completed in less than two minutes or with excessive missing data were discarded. Overall, 1.2% of the data was missing, and missing data was imputed using a nearest-neighbors weighted mean approach as implemented in the rfImpute() function in the randomForest package for R, using a response variable defined by all possible combinations of recorded sex, gender identity (cisgender or non-cisgender), and sexual orientation (heterosexual or non-heterosexual). This has the effect of imputing missing data with heavier weights on individuals that share the same combinations of recorded sex and stakeholder status as the subject of the imputation. A total of *N = 768* responses were used in the subsequent analyses.

#### Regression and false discovery rate correction

**Fig 1:**
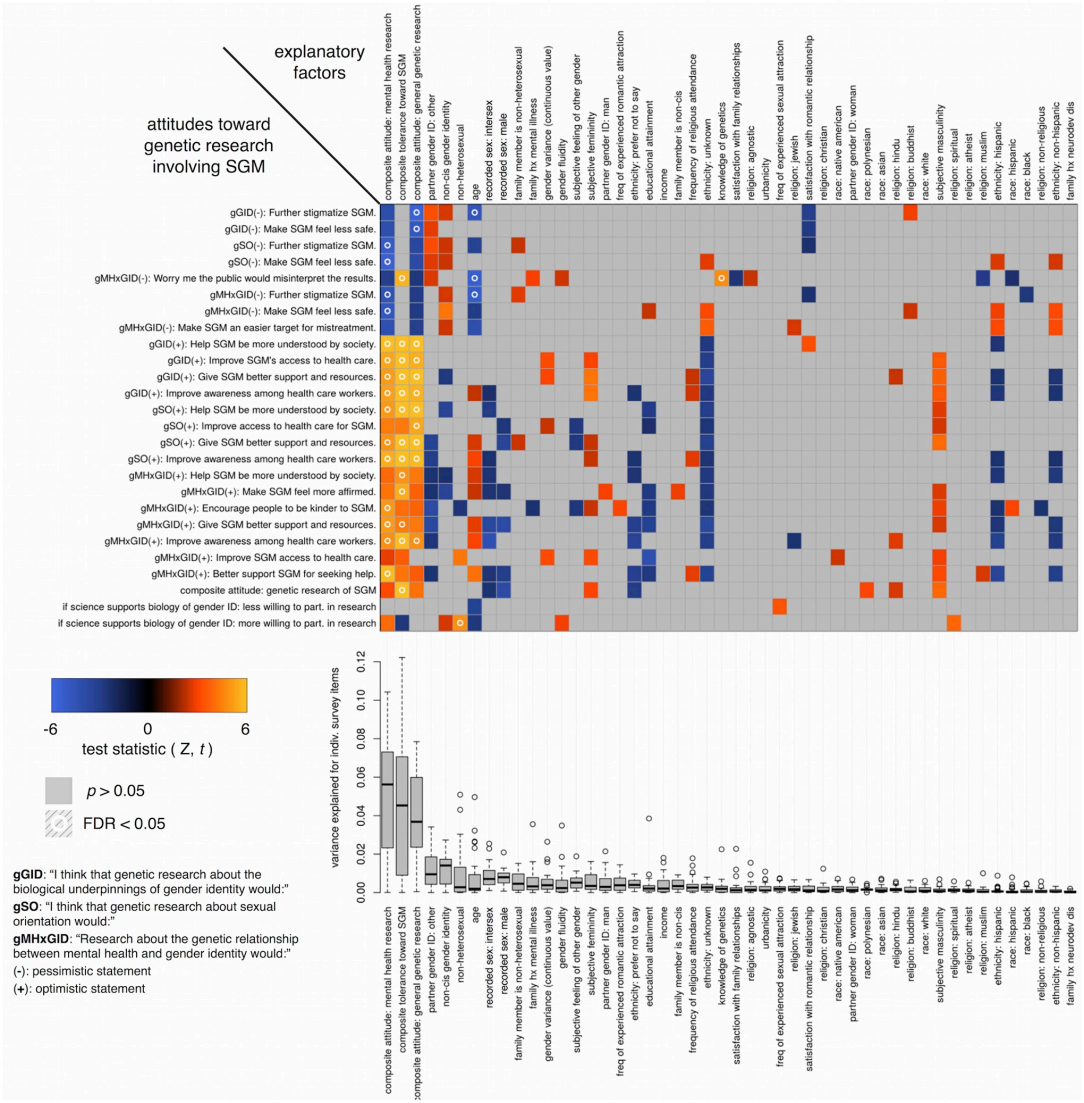
Survey items relation to genetic attitudes. The statistical associations between survey items related to attitudes toward genetic research involving SGM (rows) and explanatory factors (columns). Explanatory factors (columns) are ordered by the mean variance explained (boxplots below) over the considered survey items. Negative associations are in blue, positive associations are in orange, and non-significant associations are in gray. Boxes with a circle represent associations that survive multiple testing correction. A table of the top panel full Z-statistics is available in S2 File.

The imputed tabular survey data was used to test the association of individual survey items, as well as a composite score (a linear combination of all these items weighted by 1 if it represented an optimistic statement and -1 if it represented a pessimistic statement) with the explanatory factors shown in Fig 1. Generalized linear models using the glm function in R were used to carry out these tests in a multiple linear regression framework, with quasi-Poisson regression family=“quasipoisson” for Likert-scale items, binomial regression family=“binomial” for binary items, and linear regression family=“gaussian” for the composite score. The test statistics shown in Fig 1 are those from models where each row was modeled as a function of all columns included together. Correcting for multiple testing was performed using the Benjamini-Yekutieli procedure [19] for false discovery rate (FDR), which is valid under arbitrary assumptions, including correlated hypotheses. In addition, boxplots in Fig 1 show variance explained for each column variable across each row survey item or composite score.

#### Power

A key question is whether our survey sample is sufficiently powered to detect whether stakeholder status is a significant contributor to opinions on genetic research involving SGM. We used the pwr.r.test() function from the pwr package for R to calculate power given the sample size and correlations, which yielded estimates of 0.8-0.98 assuming an effect size (Pearson’s *r* for this test) of 0.1-0.14, suggested by the observed 1-2% variance explained by non-cisgender identity (see boxplots in Fig 1).

## Results

The sample characteristics of our participants are described in Table 2. We identified non-cisgender individuals as a participant who did not exclusively identify as cisgender and selected at least one of the other gender identities. We identified non-heterosexual individuals by a participant who did not exclusively identify as heterosexual and selected at least one of the other sexual orientations. The sample shows a significant enrichment of the LGBTQ+ community: 11% (*N = 85*) vs. 0.5% nationally (P < 0.001, *χ*^2^ = 1653.2) reporting non-cisgender identity and 35% (*N = 270*) vs. 4.1% nationally (P < 0.001, *χ*^2^ = 1812.9) reporting non-heterosexual orientation. In addition, stakeholder status was significantly (P < 0.001, W = 54642, Wilcoxon test) and positively associated with the volume of feedback in the free text fields (see Table 4 for representative examples).

**Table 4:** Representative excerpts taken from free text responses. Participants were given the option to answer all, any, or none of the questions. We selected responses that were representative of a frequently expressed thought, or that we found to be particularly compelling.

Question	Excerpt
What would you like to learn from genetic research on sexuality, gender variance, and mental health?	Genetic research offers nothing of value other than a eugenics movement in regards to the LGBTQIA community. As an intersex person, I’m well aware that they’re already trying to prevent people like me from being born with medications and various tests administered in pregnancy.
	Are the genetics of sexuality and gender variance directly correlated to the genetics of mental health? I believe research shows a higher prevalence of mental health issues in people with sexuality/gender variances but is that due to a gene that covaries with mental health and sexuality/gender issues or is it due to not fitting in with societal norms, lower self esteem, lack of acceptance by many major religions, and societal groups?
	Any research into physical differences between people who label themselves with different sexuality and gender identities would never take into full consideration all the environmental factors that have influenced a person’s sexuality, gender identity, and how they choose to label and express their identities.
	I don’t have a specific thing I would like to learn, but moreover, I think it’s a very interesting topic. As a transgender person, I would love to see genetic links or anything research has to offer. It’s just an interesting topic. And the field has potential, so long as it’s not used to abuse people but merely doing unbiased research.
	I am interested in the possible connections between these things, but I worry that the public is not ready. I think research in this area should be focused on ways to improve healthcare for gender non-conforming people and not on confirming whether or not someone is a certain identity.
	I’d like to know how many gene combinations can be involved in the manifestation of sexuality, gender and mental health since these are all such nuanced topics with subtle variations. I wonder if genes have a significant impact on determining these factors or not.
	Most of the trans people I know- myself included- are somewhere on the autism spectrum. Is there any genetic or other kind of link that would explain the seemingly higher rates of autistic trans people?
How can we be more sensitive and/or inclusive in our work?	Be careful how the results are framed/presented. They could easily be reframed by opposing activists to make it look like being LGBT (or not being cisgender) could leads to mental health problems.
	A lot of genetic research ignores the complex interactions between genes, culture and development. As a result there’s an inadvertent slip to deterministic interpretations that can provide some relief from discrimination by encouraging more tolerance of GSM (they were born that way) but can also increase intolerance and a search for “cures” or the legitimation of eugenic ideologies.
	Just continue being aware that how people identify is a personal concept and just know that it can be an emotional ground to tread
	Be careful of racial/Western bias in your idea of gender and gender non-conformity.
	Make sure that people understand that there are a variety of views on these topics based on how we were grown and raised, and no matter the research, our views have been placed.
	I really think that someone that identifies as a SGM or other controversial labels could best tell you how to be more sensitive but it’s very important to listen to every aspect of what they have to say.
	Be open to the idea that gender variance is a debatable idea and might be a social construct.
	Please consider involving a gender-studies researcher (preferably a transgender one) in the entirety of your research process, if you insist upon researching about genetics + gender + mental health. A social scientist could provide very valuable insights, especially one whose field of expertise lies in historical scientific malpractice. Additionally, please try to involve at least one transgender and at least one non-straight person in your research process. A token person cannot speak for everyone, of course, and will not catch every error. But having at least one member of the community which you are studying is better than having none!
If there were questions you did not feel comfortable answering, can you help us to understand why?	A lot of the issues have personal ties that I’m still reconciling to my Catholic faith
	It made me very uncomfortable reading all of the gender ‘options’… Bisexual and trans is too much as is, but there were way more listed.
	I felt comfortable with all of the questions, but I was unsure/uninformed on many questions
Is there anything else that you would like us to know?	Consult resources on inclusiveness within the populations you intend to target in order to better achieve your research aims.
	Stop giving imaginary genders/orientations legitimacy. Stop trying to force everyone to include their pronouns, it’s insulting. It’s like if someone went up to you and asked if you are a man or woman. It’s incredibly rude.
	Although I am confident in my gender identity, if there were a “trans” gene and I was to discover I don’t have it, it would make me worried I made a mistake. I have already worked very hard to be comfortable with my identity, and I don’t think I need genetic proof to know who I am.

### Key drivers of attitudes toward genetic research on SGM

We found that attitudes toward genetic research specifically on SGM are most strongly predicted by broader attitudes toward mental health and genetic research in general (Fig 1). Participants who expressed reservations about mental health or genetic research in general were significantly more likely to express reservations or pessimism about genetic research on SGM. Similarly, participants who endorsed the value of genetic or mental health research generally were also more likely to view genetic research on SGM positively.

We used a combination of survey items (see Table 3) to build a composite non- heterosexuality and gender variance tolerance score. This composite tolerance score was significantly and positively associated with attitudes on genetic research involving SGM, meaning that those who were more accepting of SGM and associated behaviors were more likely to view genetic research involving SGM favorably. A notable exception to this pattern was observed on the item that expressed concern that the public would misinterpret genetic findings involving SGM, where tolerance was significantly (FDR < 0.05, Z = 4.8) associated with positive endorsement of the concern (Fig 1). This item also showed significant associations with age (FDR < 0.05, Z = -4.6), and objectively measured knowledge of genetics (FDR < 0.05, Z = 4.1), suggesting that concern about public misinterpretation of the results of genetic research on SGM is expressed most strongly among younger participants who are more tolerant and more conversant in genetic concepts.

To a lesser extent, stakeholder status related to non-cisgender identity (for the participant themselves and/or for their romantic partner) trends toward concern about genetic research involving SGM. None of these associations survived correction for multiple hypothesis testing. Closer examination of these associations revealed that extreme heterogeneity of opinion within stakeholder groups, rather than small effect sizes, is the key factor preventing stronger associations of attitudes with stakeholder status. In any case, power analyses (see Methods) suggest that our sample is sufficiently powered to detect stakeholder effects when they exist.

Despite traces of concern about genetic research on SGM, both non-cisgender and non-heterosexual participants indicated that they would be more likely to participate in genetic research involving SGM if science demonstrates a biological link for gender identity (only the non-heterosexual association survives multiple testing correction at FDR < 0.05, Z = 4.0).

Finally, the age of the participant was a significant explanatory factor for attitudes on genetic research involving SGM, with younger participants trending more pessimistic and older participants trending more optimistic.

### Gender variance and its relationship to tolerance and family history of neuropsychiatric conditions

**Fig 2:**
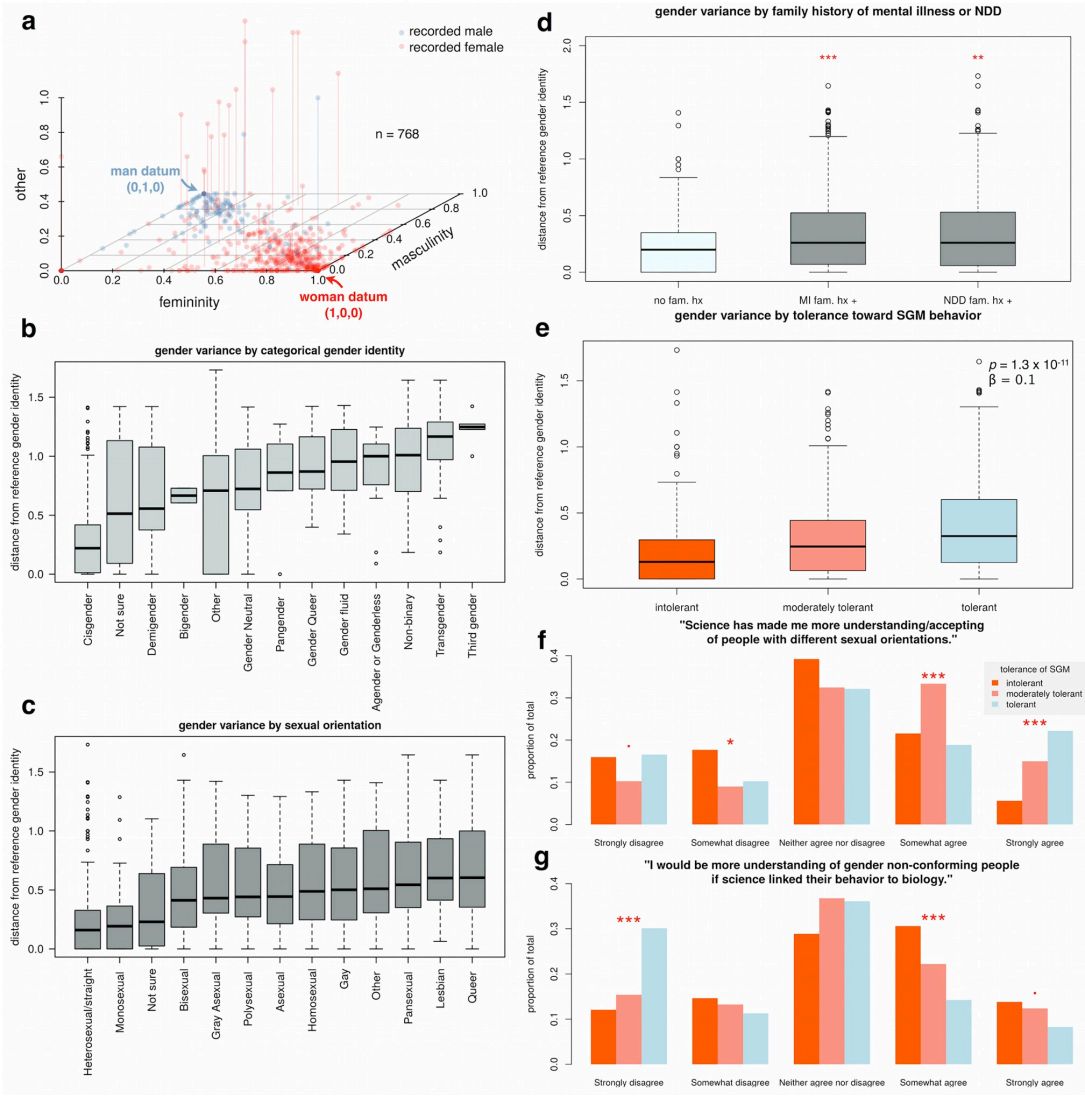
Gender variance and tolerance. A three-dimensional gender identity space (a) was used to calculate a scalar-valued gender variance score, which is the Euclidean distance from the expected reference gender identity (woman or man datum) based on the recorded sex of the participant. Scalar gender variance corresponded in a largely expected way to categorical descriptors of gender identity (b), and sexual orientation (c). We found that participants with a family history of either mental illness or neurodevelopmental disorders (NDD) showed greater gender variance (d). Those who indicated greater tolerance of SGM also showed greater gender variance (e). When reflecting on previous shifts in personal acceptance of non-heterosexual orientations, participants with higher current levels of tolerance attributed some of that shift to science (f). When considering future attitudes toward gender non-conformity, those who currently display the lowest levels of tolerance were significantly more likely to endorse science as a potential avenue toward increased personal acceptance (g). Significance key: . = p < 0.1; * = p < 0.05; ** = p < 0.01; *** = p < 0.001.

Our survey included a novel means for participants to describe their gender identity in a continuous fashion. This three-dimensional gender space (Fig 2a) allowed us to create a continuous measure of gender variance (see Methods), which varied both by categorical gender identity (Fig 2b) and categorical sexual orientation (Fig 2c). By calculating this continuous measure of gender variance for cisgender participants as well as those with non-cisgender identities, we were able to include the entirety of our sample in examining gender variance as it relates to other variables measured (Fig 2d and 2e). In doing so, we found that participants who endorsed either a family history of mental illness or neurodevelopmental disorders (Fig 2d) showed significantly greater gender variance than those who did not (P < 0.01, W = 40486, Wilcoxon test), in agreement with previous findings [20] [21]. In addition, we also found that increasing tolerance toward SGM and associated behaviors was associated with increasing levels of gender variance of the participant (Fig 2e, P < 0.001, *β* = 0.1, t = 6.9, linear model).

### The impact of science on the views of low-tolerance participants

Those who most strongly endorse science as a past contributor to their personal increased acceptance of non-heterosexuals are among the most tolerant currently (Fig 2f). Specifically, among those who “strongly agree” that science has made them more accepting, there is a monotonically increasing trend of tolerance (P < 0.001, *β* = 0.7, Z = 5.1, binomial generalized linear model). Among those who “somewhat agree”, those currently showing moderate tolerance are over-represented (P < 0.001, *β* = 0.7, Z = 3.9, binomial generalized linear model), which may indicate a process of transition from low tolerance to high tolerance.

Those who are currently least tolerant of SGM endorse science as a potential avenue for their own increased acceptance of gender non-conforming individuals (Fig 2g). These lower-tolerance groups were significantly over-represented in the “strongly agree” group (P < 0.05, *β* = -0.28, Z = -2.0, binomial generalized linear model), and in the “somewhat agree” group (P < 0.001, *beta* = -0.5, Z = -4.5, binomial generalized linear model).

### Direct statements from stakeholders

To supplement our objective, quantitative analysis of survey participant attitudes, we included representative excerpts of stakeholder feedback from the open-text fields at the end of our survey. These statements, included in Table 4, provide important insight into the necessary considerations when conducting genetic research involving SGM.

## Discussion

This study provides the first systematic look at community attitudes toward genetic research at the intersection of sexual orientation, gender identity, and mental health. Past genetic research on sexual and gender minorities (SGM) has often proceeded in the absence of input from stakeholders, and as the field accelerates, it is vital to devote time and effort to engage stakeholders as partners rather than subjects. Our findings suggest that the key predictors of attitudes toward genetic research specifically on SGM are 1) general attitudes toward genetic and mental health research 2) tolerance of SGM and associated behaviors and 3) age of the participant. Importantly, non-cisgender stakeholder status showed a detectable, but ultimately after FDR correction not statistically significant association with pessimism toward genetic research on SGM. Despite these concerns, our findings provide evidence suggesting that stakeholders are willing to engage with genetic researchers and that trust may be earned through that engagement.

A central point of discussion is the increased prevalence of neuropsychiatric conditions within the LGBTQ+ community and how these stakeholders feel regarding genetic research at this intersection. Our data showed that regardless of stakeholder status, the most prominent predictors of attitudes toward SGM genetic research specifically are their general attitudes towards genetic research and mental health research. Non-cisgender identity was to a lesser extent a predictor of these attitudes, but non-heterosexual identity did not achieve significance. Because there is evidence that stakeholder status influences how this line of research is viewed, it is important to emphasize clearly that non-heterosexuality and gender variance are not neuropsychiatric conditions, despite having been pathologized in the past. Homosexuality was removed from the Diagnostics and Statistics Manual (DSM) in 1973 [22] and Gender Identity Disorder [23] was removed in 2013 with the publication of DSM-V [24]. This most recent DSM does include gender dysphoria, which is the diagnosis commonly required in order for transgender individuals to have gender-affirming medical care. Our data showed an association between reported family history of a mental illness or a neurodevelopmental disorder and higher gender variance (this was including both cisgender and non-cisgender participants). However, this finding does not necessarily endorse a genetic relationship between the two, and a likely confounding variable is the degree of openness by the participant when asked these sensitive questions.

Although stakeholders are the primary focal point of this study, it was vital to include cisgender and heterosexual participants, because many of the fears and concerns on the part of stakeholders have to do with how the findings of genetic and other scientific research are received by the broader public. Encouragingly, we found that scientific advancement was reported as a potential pathway toward greater personal acceptance by participants who also reported the lowest levels of current tolerance of SGM. In other words, although some stakeholders reported fear of greater stigma and persecution in the face of genetic research on SGM, those would-be persecutors reported that they would be more understanding of SGM if science provided a biological basis for behaviors and identities they don’t currently understand.

### Language regarding sex

The use of the term “recorded sex” instead of “natal sex” or “assigned sex” primarily arose from survey feedback and interactions with our community advisory council. These interactions suggested valid objections to both “natal sex” and “assigned sex”. Recorded sex, with its emphasis on the generation of a vital record, i.e., the birth certificate, is our attempt at harmonizing past genetic and other biomedical research with the inclusive, sensitive language that is appropriate for a modern and complex understanding of sex. Although assigned sex is becoming more widely adopted in clinical practice, with the DSM-5 using the term in their language regarding gender dysphoria [24], further research and consideration are needed to develop language that is appropriate in a biological research context and that is not problematic from the perspective of any gender identity.

### Limitations of this study

This study is a first important step in the engagement of the SGM stakeholder community in genetic research. Consequently, it is important to consider the limitations of our sample and design, so that results are not over-interpreted. First, our sample skews young, white, recorded female, and highly educated (when compared to national demographics). If this study were replicated in other, more ethnically diverse locations in the U.S., it is possible that some conclusions would be influenced. There are sub-threshold trends in our data that suggest that racial and ethnic minorities are more skeptical of research in general than the white majority of our sample. Despite this limitation, our sample is likely representative of the “samples of convenience” that are often the norm in current genetic research. Secondly, some of our working variables, including the tolerance indicator and the gender variance scale, should be seen as derived composite variables that represent tendencies in attitudes over a collection of related questions. These are not “scales”: they have not been normed and their psychometric properties have not been subjected to an in-depth investigation.

### Recommendations for genetic researchers

After synthesizing the survey results, the open-text feedback, and interactions that have resulted through re-contact of survey participants who volunteered for follow-up communication, a number of recommendations have emerged for scientists interested in pursuing research in this area. A common concern was the connection between the eugenics movement, medical research, and SGM. The eugenics movement rationalized abhorrent practices such as forced sterilization, psychiatric institutionalization, and immigration restriction based on traits or identities deemed undesirable by the movement [25], including the LGBTQ+ community. Given the often intersecting history between psychiatry and the eugenics movement, it is incumbent on researchers to plan, execute, and disseminate research in a way that ensures that the basic human rights of SGM are preserved and history is not repeated. The following recommendations are given in the hope of helping researchers achieve this higher standard of more responsible and considerate research.

First, we recommend that all genetic research projects involving SGM have a community advisory council (CAC) composed of stakeholders representing a variety of gender identities and sexual orientations. A CAC can provide input at the study design phase, giving important insight into research questions that are meaningful from a stakeholder perspective. CAC members can also give feedback during the manuscript preparation phase, so that a proper balance is struck between scientific accuracy and considerate messaging.

Second, we recommend that a section of the lab or consortium website be produced, written for a lay audience, that gets out in front of sensitive issues and that clearly answers questions regarding the research motivation and expected results. This website should link to peer-reviewed literature that provides supporting information. The website should also provide contact information for those wishing to express their feedback. Two example websites can be found at http://gender.devgenes.org and https://geneticsexbehavior.info.

Third, publication of results should follow careful preparation of messaging, ideally in the form of a press kit prepared in collaboration with journalists or public relations professionals. One of the main findings of the current study was clear concern that the public would easily misinterpret results of genetic research on SGM. It is therefore incumbent on researchers in this area to not only prepare manuscripts for a scientific audience, but also clear and concise takeaways for the broader public. This should not be left to chance.

Finally, diversity within a scientific team is a tremendous asset. Although there is nothing that would prevent cisgender, heterosexual scientists from performing rigorous and sensitive research in this area, the inclusion of team members who have a personal investment can provide invaluable perspective throughout the research process. It is not clear whether or how this diversity should be signaled, both within scientific circles and to the general public. Such signaling might provide increased credibility, but it also runs the risk of tokenizing individuals. Representation of stakeholder groups among the scientific team should, however, not be misinterpreted as a “blank check” to pursue research in this area without seeking broader, more systematic stakeholder input. Our findings speak to the heterogeneity of perspectives within these groups, and stakeholder scientists are unlikely to adequately capture all the relevant considerations by themselves.

## Conclusion

Our survey sample, highly enriched for LGBTQ+ community members, revealed that attitudes toward genetic research involving SGM are driven by a variety of factors, primarily by general attitudes toward research broadly, as well as tolerance of SGM. To a lesser extent, stakeholder identities were also associated with attitudes toward this area of research, both in optimistic and pessimistic ways. It is important to note that these attitudes were heterogeneous across and within different stakeholder groups. Our data do not support monolithic, cohesive, homogeneous attitudes among stakeholders. Rather, our findings support sexual orientation and gender identity as important but not uniquely decisive factors in how individuals feel about research on one aspect of their identity. We hope these results are understood not as an unequivocal endorsement of this line of research, but instead as a call for engagement and partnership between experts and stakeholders in navigating this challenging frontier.

## Supporting information

S1 File Survey. PDF of Qualtrics survey.
(PDF)

S2 File Fig 1 test statistics. Top panel of Fig 1 with all Z-statistics.
(XLSX)
