# Supplementary material for: Community attitudes on genetic research of gender identity, sexual orientation, and mental health
Source: PLoS One. 2020 Jul 8;15(7):e0235608. doi: 10.1371/journal.pone.0235608 (PMC7343141; doi:10.1371/journal.pone.0235608)
Supplement: S1 File — PDF of Qualtrics survey. (PDF) [file pone.0235608.s001.pdf]

Gender, Sexuality and Neurodevelopment Attitudes Survey

Start of Block: Consent Letter

Q51 We invite you to participate in a research study being conducted by investigators from The University of Iowa. The purpose of the study is to better understand the attitudes that people hold toward gender, sexuality, neurodevelopment, mental health, and how genetic research might play a role in each of these topics. Approximately 5,000 individuals over the age of 18 will participate in this study at the University of Iowa and surrounding areas.

If you agree to participate, we would like you to fill out a brief online survey, with questions that ask about your gender identity/expression and sexual orientation, as well as your attitudes toward these subjects generally. There will also be questions asking how you feel about genetic research in a variety of situations. We understand that some of these questions may be sensitive in nature; you may choose not to answer questions if you wish.

If you do not wish to provide your contact information, your answers will be kept completely anonymous and we will not recontact you for any reason. If you do wish to provide your contact information at the end of the survey in order to hear about future research opportunities with our lab, we will keep this information confidential. You will be assigned a participant ID number, and your survey answers will be tied to this number rather than to your name or contact information. Only approved members of our research team will have access to your contact information, as well as federal regulatory agencies and the University of Iowa Institutional Review Board (a committee that reviews and approves research studies) if they inspect and copy records pertaining to this research. If we write a report using the results of this survey, we will do so in such a way that you cannot be identified.

There are no known risks from taking part in this study, and you will not benefit personally. However, we hope that others may benefit in the future from what we learn as a result of this study. There are no costs for being in this research study and you will not be paid for being in this research study.

Taking part in this research study is completely voluntary. If you do not wish to participate in this study, simply close your browser and no information will be collected from you. You may choose to stop participating at any time during the survey; any answers entered up to that point would be automatically saved - anonymously.

If you have any questions about the research study itself, please contact Natalie Pottschmidt at info@devgenes.org or (319) 335-8882. If you experience a research-related injury, please contact Jacob Michaelson, (319) 335-8066. If you have questions about the rights of research subjects, please contact the Human Subjects Office, 105 Hardin Library for the Health Sciences, 600 Newton Rd, The University of Iowa, Iowa City, IA 52242-1098, (319) 335-6564, or e-mail irb@uiowa.edu.

Thank you very much for your consideration! To indicate your willingness to participate in this study, please continue to the survey by clicking the “Next” button below.

End of Block: Consent Letter

Start of Block: General Demographics

Q29 Please enter your current **age** in years.

____

Q30 Which of the following best describes your **race**?

- ▢ White or Caucasian (1)
- ▢ Hispanic or Latino (2)
- ▢ Black or African American (3)
- ▢ Native American or Alaskan Native (4)
- ▢ Asian (5)
- ▢ Native Hawaiian or Other Pacific Islander (6)
- ▢ Not sure (8)
- ▢ Prefer not to say (9)
- ▢ Other (describe) (7) ____

Q52 Which of the following best describes your **ethnicity**?

- ☐ Hispanic or Latino (1)
- ☐ Not Hispanic or Latino (3)
- ☐ Not sure (4)
- ☐ Prefer not to say (5)

Q31 Which of the following best describes your **highest level of educational attainment**?

- ○ Some high school (1)
- ○ High school degree (2)
- ○ Some college, no degree (3)
- ○ Associate degree (4)
- ○ Bachelor's degree (5)
- ○ Master's degree (6)
- ○ Professional degree (7)
- ○ Doctorate (8)
- ○ Not sure (10)
- ○ Prefer not to say (11)
- ○ Other (describe) (9) ____

Q53 Which of the following best describes your **annual income**?

- ○ Less than $14,999 (1)
- ○ $15,000-$24,999 (2)
- ○ $25,000-$34,999 (3)
- ○ $35,000-$49,999 (4)
- ○ $50,000-$74,999 (5)
- ○ $75,000-$99,999 (6)
- ○ $100,000-$149,999 (7)
- ○ $150,000-$199,999 (8)
- ○ More than $200,000 (9)

Q27 Which of the following best describes your **religious/spiritual affiliation**? Select all that apply.

- Christian (1)
- Jewish (2)
- Hindu (3)
- Muslim (4)
- Buddhist (5)
- Atheist (6)
- Agnostic (7)
- Non-religious (8)
- Spiritual (9)
- Not sure (11)
- Prefer not to say (12)
- Other (describe) (10) ____

Q32 How often do you attend **religious services**?

- ◯ Not at all (1)
- ◯ Sometimes, but not regularly (2)
- ◯ Once a week (3)
- ◯ More than once a week (4)
- ◯ Prefer not to say (5)

Q28 Which of the following best describes the area **where you live**?

- ◯ Suburban (1)
- ◯ Rural (2)
- ◯ Urban (3)
- ◯ Not sure (5)
- ◯ Prefer not to say (6)
- ◯ Other (describe) (4) ____

Q50 Have you or anyone in your family been diagnosed with:

	Yes (1)	No (2)	Not sure (3)	Prefer not to say (4)
**Cancer** (1)	○	○	○	○
**Autoimmune Disease** (For example: multiple sclerosis, rheumatoid arthritis, type 1 diabetes) (2)	○	○	○	○
**Cardiovascular Disease** (For example: coronary heart disease, deep vein thrombosis, congenital heart disease, etc.) (3)	○	○	○	○
**Mental Health Disorder** (For example: major depression, generalized anxiety disorder, bipolar disorder, schizophrenia, etc.) (4)	○	○	○	○
**Neurodevelopmental Disorder** (For example: autism spectrum disorder, intellectual disability, attention deficit disorder, etc.) (5)	○	○	○	○
**Substance Use Disorder** (For example: alcohol, illicit drugs, etc.) (6)	○	○	○	○

Q55 Do you have an immediate family member who is **not heterosexual**? In other words, do you have an immediate family member who is, for example, gay, lesbian, or bisexual?

- Yes (1)
- No (2)
- Not sure (3)
- Prefer not to say (4)

Q54 Do you have an immediate family member who displays **gender variance**? In other words, do you have an immediate family member who dresses or acts in a way that is a clear departure from their biological sex.

- Yes (1)
- No (2)
- Not sure (3)
- Prefer not to say (4)

End of Block: General Demographics

Start of Block: Knowledge of Genetics

Q32 Please respond to the following statements by indicating your level of agreement/disagreement.

	Strongly disagree (6)	Somewhat disagree (7)	Neither agree nor disagree (8)	Somewhat agree (9)	Strongly agree (10)	Not sure (11)
You can see a gene with the naked eye. (1)	○	○	○	○	○	○
Genes come from biological parents. (2)	○	○	○	○	○	○
Diseases that run in the family are related to genes. (3)	○	○	○	○	○	○
A gene is a type of cell. (4)	○	○	○	○	○	○
It has been suggested that a person has 22,000 genes. (5)	○	○	○	○	○	○

End of Block: Knowledge of Genetics

Start of Block: Attitudes Towards Genetic Research

Q36 Please respond to the following statements by indicating your level of agreement/disagreement.

	Strongly disagree (3)	Somewhat disagree (4)	Neither agree nor disagree (5)	Somewhat agree (6)	Strongly agree (7)	Not sure (8)
Genetic research is helpful for the treatment of diseases. (1)	○	○	○	○	○	○
Genetic research will help others in the future. (2)	○	○	○	○	○	○
I am open to participating in genetic research. (3)	○	○	○	○	○	○
Genetic research does more harm than good. (4)	○	○	○	○	○	○
I am worried that genetic research will compromise my privacy. (5)	○	○	○	○	○	○
I am worried that information from genetic research could be used against me. (6)	○	○	○	○	○	○
I am afraid I could be discriminated against because of	○	○	○	○	○	○

genetic information. (7)

Q37 Information learned from research on my own genetic makeup could:

	Strongly disagree (3)	Somewhat disagree (4)	Neither agree nor disagree (5)	Somewhat agree (6)	Strongly agree (7)	Not sure (8)
Influence my decision to have a child. (1)	o	o	o	o	o	o
Change decisions regarding my healthcare. (2)	o	o	o	o	o	o
Change what career I choose. (3)	o	o	o	o	o	o
Alter my retirement plan. (4)	o	o	o	o	o	o
Influence my insurance coverage options. (5)	o	o	o	o	o	o

Q48 I think that it is okay to use genetic technology to:

	Strongly disagree (3)	Somewhat disagree (4)	Neither agree nor disagree (5)	Somewhat agree (6)	Strongly agree (7)	Not sure (8)
Choose the sex of a child. (1)	○	○	○	○	○	○
Reduce or eliminate the risk for physical health problems. (5)	○	○	○	○	○	○
Reduce or eliminate the risk for mental health or neurodevelopmental problems. (2)	○	○	○	○	○	○
Influence appearance of an unborn child. (3)	○	○	○	○	○	○
Influence personality or temperament of an unborn child. (4)	○	○	○	○	○	○
Influence the appearance of an adult. (6)	○	○	○	○	○	○

Q50 Please respond to the following statements by indicating your level of agreement/disagreement.

	Strongly disagree (3)	Somewhat disagree (4)	Neither agree nor disagree (5)	Somewhat agree (6)	Strongly agree (7)	Not sure (8)
Making changes to a person's genome is okay. (1)	○	○	○	○	○	○
Gene editing is unethical. (2)	○	○	○	○	○	○
I feel okay about genetically modified organisms (e.g., designer crops). (3)	○	○	○	○	○	○
Terminating a pregnancy based on the results of a genetic test is acceptable. (4)	○	○	○	○	○	○

End of Block: Attitudes Towards Genetic Research

Start of Block: Attitudes Towards Mental Health Research

Q54 Please respond to the following statements by indicating your level of agreement/disagreement.

	Strongly disagree (2)	Somewhat disagree (3)	Neither agree nor disagree (4)	Somewhat agree (5)	Strongly agree (6)	Not sure (7)
Research on mental health makes treatment better. (1)	o	o	o	o	o	o
Mental health research will help others in the future. (2)	o	o	o	o	o	o
I am open to participating in mental health research. (3)	o	o	o	o	o	o
Research on mental health does more harm than good. (4)	o	o	o	o	o	o
I am worried that information from research on my mental health could be used against me in the future. (5)	o	o	o	o	o	o

Q55 Information I learn from research about my mental health could:

	Strongly disagree (3)	Somewhat disagree (4)	Neither agree nor disagree (5)	Somewhat agree (6)	Strongly agree (7)	Not sure (8)
Influence my decision to have a child. (1)	o	o	o	o	o	o
Change decisions regarding my healthcare. (2)	o	o	o	o	o	o
Change what career I choose. (3)	o	o	o	o	o	o
Alter my retirement plan. (4)	o	o	o	o	o	o
Influence my insurance coverage options. (5)	o	o	o	o	o	o

End of Block: Attitudes Towards Mental Health Research

Start of Block: Gender and Sexuality Demographics

Q64 What are your **gender pronouns** (what pronouns do you prefer when others speak about you)?

- ☐ She, her, hers (1)
- ☐ He, him, his (2)
- ☐ They, them, their/theirs (3)
- ☐ Ze, Hir/Zir, Hirs/Zirs (4)
- ☐ Not sure (6)
- ☐ Prefer not to say (7)
- ☐ Other (describe) (5) ____

Q65 Select your **sex assigned at birth**, on your **original birth certificate** (we understand you may not describe yourself in this way now, but this information is helpful for our research).

- ○ Male (1)
- ○ Female (2)
- ○ Intersex (3)
- ○ Not sure (5)
- ○ Prefer not to say (6)
- ○ Other (describe) (4) ____

Q61
What gender or genders do you identify as? We understand that this may vary, but please describe this on average if at all possible.

▢ Man (1)

▢ Woman (2)

▢ Third Gender (3)

▢ Does not apply (4)

▢ Not sure (5)

▢ Prefer not to say (6)

▢ Other (describe) (7) ____

Q66
Place the sliders below in a configuration that best describes your **gender identity.** We understand that some people are gender fluid and that this question may not fully capture that. Gender fluidity will be covered in a separate question.

	Not at all	Extremely	Not Applicable
Masculine ()			
Feminine ()			
Other gender (describe) ()			

Q67
Select the labels, if any, that you find useful in describing your current gender (**gender identity**). This could be consistent with, or different than, the sex you were assigned at birth.

- ☐ Cisgender (Someone who identifies with a gender that is consistent with their assigned sex at birth) (1)
- ☐ Transgender (Someone who does not identify or exclusively identify with their sex assigned at birth) (2)
- ☐ Non-binary (An umbrella term for all genders other than female/male or woman/man) (3)
- ☐ Demigender (Someone who feels a partial, but not a full, connection to a particular gender identity or just to the concept of gender) (5)
- ☐ Gender fluid (Someone with a changing or “fluid” gender identity) (4)
- ☐ Third gender (Someone who identifies as neither male nor female) (6)
- ☐ Agender or Genderless (Someone who sees themselves as not having a gender) (7)
- ☐ Gender Neutral (Someone who acknowledges gender, but does not identify with a gender construct) (12)
- ☐ Pangender (Someone who identifies as being more than one gender) (8)
- ☐ Bigender (Someone whose sense of personal identity encompasses two genders) (15)
- ☐ Gender Queer (Someone who does not subscribe to conventional gender distinctions but identifies with neither, both, or a combination of male and female genders) (17)
- ☐ Prefer not to say (10)

- ☐ Not sure (11)
- ☐ Other (please include your definition) (9) ______________________________

Q42 On a scale of "stays the same" to "changes frequently", how constant is your **gender expression** over time?

	Stays the same	Changes sometimes	Changes frequently	Not Applicable
Adjust the slider to reflect how your **gender expression** changes over time. ()				

Q38
Select the gender(s) of **your current and past romantic partner(s)**. This should reflect what you consider to be your general tendency in preference. We understand that this may vary, but please describe this on average if at all possible.

- ☐ Man (1)
- ☐ Woman (2)
- ☐ Third Gender (3)
- ☐ Does not apply (4)
- ☐ Not sure (5)
- ☐ Prefer not to say (6)
- ☐ Other (describe): (7) ______________________________

Q37 In general, I feel **sexual attraction** toward others:

- Never (1)
- Infrequently (2)
- Routinely (3)
- Constantly (4)

Q56 In general, I feel **romantic attraction** toward others:

- Never (1)
- Infrequently (2)
- Routinely (3)
- Constantly (4)

Q43 Please select any terms you find useful in describing your current **sexual orientation** (your identity in relation to what gender(s) you are attracted to emotionally, romantically, and/or sexually).

- ▢ Gay (1)
- ▢ Lesbian (2)
- ▢ Bisexual (3)
- ▢ Heterosexual/straight (8)
- ▢ Homosexual (9)
- ▢ Asexual (Someone who does not feel sexual attraction towards other people) (4)
- ▢ Pansexual (Someone who is attracted to many/any gender(s)) (5)
- ▢ Queer (Someone who is not cisgender and/or heterosexual) (6)
- ▢ Monosexual (Someone who has a sexual attraction toward one gender only) (7)
- ▢ Gray Asexual (Someone who may only experience sexual attraction on occasion; the gray area between asexuality and sexuality) (10)
- ▢ Polysexual (Someone who is attracted to multiple genders) (11)
- ▢ Not sure (13)
- ▢ Prefer not to say (14)
- ▢ Other (describe) (12) ____

Q57 Are you satisfied with your relationships within your immediate family?

- Yes (1)
- Sometimes (4)
- No (5)
- Not sure (6)
- Prefer not to say (7)

Q58 If you are in a committed relationship, are you satisfied with that relationship?

- Yes (1)
- Sometimes (2)
- No (3)
- Not sure (4)
- Does not apply (5)
- Prefer not to say (6)

Q59 If you are not in a committed relationship, are you interested in being in one?

- ○ Yes (1)
- ○ Sometimes (2)
- ○ No (4)
- ○ Not sure (5)
- ○ Does not apply (6)
- ○ Prefer not to say (7)

End of Block: Gender and Sexuality Demographics

Start of Block: Attitudes Towards Research About Gender Identity

Q44 Please respond to the following statements by indicating your level of agreement/disagreement.

	Strongly disagree (3)	Somewhat disagree (4)	Neither agree nor disagree (5)	Somewhat agree (6)	Strongly agree (7)	Not sure (8)
How people perceive their gender (**gender identity**) is a choice. (1)	○	○	○	○	○	○
Women should only be attracted to men. (6)	○	○	○	○	○	○
It is okay that people dress in ways that don't conform with their sex assigned at birth. (2)	○	○	○	○	○	○
People are supposed to be male or female. (3)	○	○	○	○	○	○
Men should only be attracted to women. (4)	○	○	○	○	○	○
Some people do not experience sexual attraction at all. (5)	○	○	○	○	○	○

Q45
*This question relates to your opinion about research and how it impacts **sexual and gender minorities (SGM).***

I think that genetic research about the biological underpinnings of **gender identity** would:

	Strongly disagree (3)	Somewhat disagree (4)	Neither agree nor disagree (5)	Somewhat agree (6)	Strongly agree (7)	Not sure (8)
Help SGM be more understood by society. (1)	○	○	○	○	○	○
Further stigmatize SGM. (2)	○	○	○	○	○	○
Make SGM feel less safe. (3)	○	○	○	○	○	○
Improve SGM's access to health care. (4)	○	○	○	○	○	○
Give SGM better support and resources. (5)	○	○	○	○	○	○
Improve awareness among health care workers. (6)	○	○	○	○	○	○

Q46 Please respond to these statements, indicating your level of agreement or disagreement.

	Strongly disagree (3)	Somewhat disagree (4)	Neither agree nor disagree (5)	Somewhat agree (6)	Strongly agree (7)	Not sure (8)
I would be more understanding of gender non-conforming people if science linked their behavior to biology. (1)	○	○	○	○	○	○
Science has made me more understanding/accepting of people with different sexual orientations. (3)	○	○	○	○	○	○
I hope science helps us better understand gender non-conformity. (4)	○	○	○	○	○	○

Q43 *This question relates to your opinion about research and how it impacts* ***sexual and gender minorities (SGM).***

I think that genetic research about **sexual orientation** (i.e., who people are physically, emotionally and/or romantically attracted to) would:

	Strongly disagree (3)	Somewhat disagree (4)	Neither agree nor disagree (5)	Somewhat agree (6)	Strongly agree (7)	Not sure (8)
Help SGM be more understood by society. (1)	○	○	○	○	○	○
Further stigmatize SGM. (2)	○	○	○	○	○	○
Make SGM feel less safe. (3)	○	○	○	○	○	○
Improve access to health care for SGM. (4)	○	○	○	○	○	○
Give SGM better support and resources. (5)	○	○	○	○	○	○
Improve awareness among health care workers. (6)	○	○	○	○	○	○

End of Block: Attitudes Towards Research About Gender Identity

---

Start of Block: Attitudes Towards Genetic Research About Mental Health, Gender and Sexuality

Q23 *This question relates to your opinion about research and how it impacts* ***sexual and gender minorities (SGM).***

Research about the genetic relationship between **mental health** and how people perceive their gender (**gender identity**) would:

	Strongly disagree (3)	Somewhat disagree (4)	Neither agree nor disagree (5)	Somewhat agree (6)	Strongly agree (7)	Not sure (8)
Worry me the public would misinterpret the results. (10)						
Help SGM be more understood by society. (1)						
Further stigmatize SGM. (2)						
Make SGM feel more affirmed. (8)						
Encourage people to be kinder to SGM. (7)						
Make SGM feel less safe. (3)						
Make SGM an easier target for mistreatment. (9)						
Give SGM better support and resources. (5)						

Improve awareness among health care workers. (6)	○	○	○	○	○	○
Improve my access to health care. (4)	○	○	○	○	○	○
Better support SGM for seeking help. (11)	○	○	○	○	○	○

Q24 If research reveals a **connection** between certain **mental health conditions and gender non-conformity**, how would that possibility influence your willingness to participate in research on this topic?

○ More willing (1)

○ Neither more nor less willing (2)

○ Less willing (3)

○ Not sure (4)

○ Prefer not to say (5)

Q25 If research establishes a clear **biological basis for gender non-conformity**, how would that potential outcome influence your willingness to participate in research on this topic?

- ○ More willing (1)
- ○ Neither more nor less willing (2)
- ○ Less willing (3)
- ○ Not sure (4)
- ○ Prefer not to say (5)

End of Block: Attitudes Towards Genetic Research About Mental Health, Gender and Sexuality

---

Start of Block: Free-Response

Q30 What would you like to learn from genetic research on sexuality, gender variance, and mental health?

____

Q31 How can we be more sensitive and/or inclusive in our work?

____

Q32 If there were questions you did not feel comfortable answering, can you help us to understand why?

____

Q40 Is there anything else that you would like us to know?

____

End of Block: Free-Response

Start of Block: Contact Information Follow Up

Q47 In the future, I would like to be contacted about research regarding **mental health, sexuality, and gender variance**.

- Yes (1)
- No (2)

Display This Question:

If In the future, I would like to be contacted about research regarding mental health, sexuality, an... = Yes

Q48 Contact Information

- Name (1) ____
- Phone number (2) ____
- Email (3) ____

End of Block: Contact Information Follow Up
